# Substitution of Physicians’ Prescriptions

**Published:** 1903-05

**Authors:** 


Substitution of Physicians' Prescriptions.—The alarming extent to which this nefarious, nay, criminal, practice is carried on in Texas was partially revealed during the recent session of the Legislature. Dr. Miller, of Milam, who is fully impressed with the necessity of some measures of protection of the profession and the public against this great danger, prepared a bill to meet the case. The bill was unanimously reported favorably by the Committee on Public Health in the House; but it got out, through the vigilance of the offending "substituters," and monster petitions against its passage were sent in through their paid lobbyists. The bill "died a-bornin'."

The medical profession can not afford to be indifferent to this great evil. They must arouse to a sense of the danger, and unite to have it remedied. The local societies must take notice and send in petitions to the State Association to memoralize the Governor and the law makers for relief. It is, first, a constant and hidden menace to the public health. It is a wrong and a fraud upon the unsuspecting parties who carry prescriptions to these unscrupulous compounders of medicines—these human vultures who fatten on the credulity of the public and the misery and misfortunes of the sick and afflicted; and last, it is a fraud—a swindle—an injury and a wrong upon the trusting physician who sends his patronage to these concealed swindlers. A "substituter" has the life of the patient in his hands. He can, and doubtless does, destroy many lives by putting up inferior, cheap and impure and, therefore, dangerous chemicals, when true, tried and reliable pharmaceuticals have been ordered. The doctor never knows it. The child or man or woman—dies, the doctor is dismissed and loses the patronage, influence and good will of the family and all its connections. Dr. Miller told me of a case where commercial arsenic had been substituted for a well known and valuable preparation which had been ordered, and the result was nearly a fatal termination. Thus it will be seen these hyenas have the doctor's fortune, his fame and his career in their hands, and they may and do, not infrequently, wreck all three for the sake of a paltry profit on "something just as good," of their own preparation. I'm going to the Brazos Valley Medical Association meeting at Cameron this month, and I will start the ball in motion by a resolution or memorial. I want some of the readers of the *Red Back* to read this to their associates in every society meeting, and let us get up a strong sentiment in favor of reform, so that by next meeting of the Legislature we may be organized, and get a bill passed to knock these fellows out and send some of them to the *pen*, where they all ought to be!

